# Hemodynamic predictors of negative false lumen remodeling after frozen elephant trunk for acute aortic dissection

**DOI:** 10.1007/s11748-023-01984-x

**Published:** 2023-11-10

**Authors:** Yusuke Takei, Shohei Miyazaki, Kohei Suzuki, Shunsuke Saito, Hayato Oogaki, Yuki Muraoka, Takeshi Ogasawara, Masahiro Tezuka, Ikuko Shibasaki, Hirotsugu Fukuda

**Affiliations:** 1 Department of Cardiac and Vascular Surgery, Dokkyo Medical University Graduate School of Medicine, 880 Kitakobayashi, Mibu-machi, Shimotuga-gun, Tochigi 321-0293 Japan; 2 Cardio Flow Design, Inc., Chiyoda, Tokyo, Japan; 3 Department of Radiology, Dokkyo Medical University Hospital, Mibu-machi, Tochigi Japan; 4 Mathematics and Statistics Section, Department of Fundamental Education, Dokkyo Medical University, Mibu-machi, Tochigi Japan

**Keywords:** Frozen elephant trunk, Four-dimensional flow magnetic resonance imaging, Negative false lumen remodeling

## Abstract

**Objective:**

We evaluated the blood flow within the downstream aortic false lumen after frozen elephant trunk repair for acute aortic dissection and identified hemodynamic predictors of false lumen expansion and negative false lumen remodeling using four-dimensional flow magnetic resonance imaging.

**Methods:**

Thirty-one patients (Stanford type A, *n* = 28; Stanford type B, *n* = 3) with patent false lumen who underwent frozen elephant trunk procedures for acute aortic dissection were included in this observational study. Each patient underwent computed tomography during the follow-up period and four-dimensional flow magnetic resonance imaging within 3 postoperative months. The false lumen volumetric expansion rate was calculated using computed tomography data. The direction and the rate of flow in the lower descending aortic false lumen were analyzed. Negative false lumen remodeling was defined as a volumetric increase of > 10% from the baseline volume.

**Results:**

Negative false lumen remodeling had developed in 6 of the 31 patients during the observation period. Most of the false lumen flows were biphasic during systole. The range between peak and nadir flow rates was associated with the false lumen volumetric expansion rate (*β* coefficient = 6.77; *p* < 0.01, *R*^2^ = 0.43).

**Conclusions:**

The range between peak and nadir flow rates may serve as a hemodynamic predictor of negative false lumen remodeling, enabling further treatment for patients at risk of expansion in the downstream aorta.

**Supplementary Information:**

The online version contains supplementary material available at 10.1007/s11748-023-01984-x.

## Introduction

The frozen elephant trunk (FET) procedure achieves excellent early outcomes, leads to aortic remodeling, which is defined as regression of the false lumen (FL) in the aortic arch, and reduces the need for subsequent interventions (re-intervention) compared with total arch replacement in the mid-term period [1–3]. However, most of the FL in the middle and lower thoracic aorta remains patent due to residual FL perfusion from re-entries in the downstream aorta, causing re-interventions [4, 5]. Therefore, a few reports have implied that additional endovascular treatment should be aggressively applied to promote aortic remodeling [6, 7]. This proposal is controversial because long-region stent graft implantation may induce spinal cord ischemia and pose an increased risk of adverse events. Nevertheless, this procedure may be feasible to perform in patients at risk of negative aortic remodeling, specifically the expansion of the aorta, especially in the FL region. Though several morphologic predictors based on computed tomography (CT) angiography findings have been employed to detect negative aortic remodeling [8, 9], and to the best of our knowledge, those that can be applied for further treatment are lacking. Notably, four-dimensional flow magnetic resonance imaging (4D flow MRI) has allowed us to perform functional analysis of the predictors of negative aortic remodeling [10, 11]. This study investigated the FL flow in patients who underwent FET with 4D flow MRI analysis and identified the hemodynamic predictors of FL expansion and negative FL remodeling.

## Patients and methods

### Ethical statement

This single-center observational cohort study received approval from the Institutional Review Board of Dokkyo Medical University (protocol number 27039) on July 14, 2015. The study adhered to the ethical standards outlined in the 1964 Declaration of Helsinki and its subsequent amendments. Written informed consent was obtained from all the patients, and the study excluded patients who requested withdrawal.

### Study design and overview

We enrolled patients who underwent FET for acute aortic dissection with a patent or partially thrombosed FL. All patients received optimal medical treatment under restricted blood pressure and heart rate, following the guidelines. Each patient underwent 4D flow MRI within 3 months after FET, and CT scans were conducted 6 months postoperatively and annually thereafter in our outpatient department. We focused on the aorta uncovered with the FET, especially from the distal end of the stent graft to the level of the celiac trunk (CA), i.e., Zones 4 and 5. We measured the volume of the aortic lumen (AL) and true lumen (TL) and calculated the FL volume. We evaluated the association between hemodynamic factors obtained from the 4D flow MRI analysis and FL expansion rate and classified patients into two groups: with or without negative FL remodeling. The exclusion criteria were as follows: FET for ulcer-like projection lesions, postoperative development of total FL thrombosis or ulcer-like projection lesions in the descending aorta, contraindications to MRI, and insufficient data for analyses (such as only plain CT data, short follow-up period [< 0.5 years], and 4D flow MRI data that could not be analyzed due to artifact).

### Total arch replacement with a FET

All patients underwent total arch replacement with FET using a previously reported technique [12]. Primary indications of FET for Stanford type A aortic dissection were previously mentioned [13], including complicated Stanford type B aortic dissection, which is not acceptable for endovascular surgery. We used the manufactured FET device, J Graft FROZENIX^®^ [14, 15] (Japan Lifeline Co., Ltd., Tokyo, Japan). The length of the implanted device was 9 cm, and it was inserted starting from Zone 1. The device diameter depended on the preoperative CT data of each patient. The chosen device always had a 10% smaller diameter than the proximal descending aorta where it was placed. Most of the distal ends of the devices were placed above the Th6 level.

### Volumetric measurements with three-dimensional CT angiography

Anatomical geometry was reconstructed from CT angiography by SOMATOM Sensation 64 (Siemens AG, Munich, Germany). All the CT data were analyzed by two investigators using Ziostations2 (Ziosoft, Inc., Tokyo, Japan). We followed the method given by Dohle et al. [16], in which volumetric measurements were performed manually along the aorta, targeting the area between the stent graft end and CA (Zones 4 and 5). AL and TL volumes were automatically measured, and FL volume was estimated based on the difference between the two volumes. All the volumes were documented in cubic centimeters. The FL status (patent, partially thrombosed, total thrombosed, or obliterated) was documented for the target segment. “Total thrombosed” was used to describe cases where thrombosis had reached the CA, and “obliterated” was used to describe cases where the FL had regressed and disappeared in addition to total thrombosis. “Patent” was used to describe cases where thrombosis had not progressed postoperatively, and “partially thrombosed” was used for cases of thrombosis that had not reached the point of total thrombosis. We defined lumen volume (LV) right after surgery as LV_0_, similar to AL_0_, TL_0_, and FL_0_. Additionally, we defined each LV at the time of the last measurement as LV_1_, including AL_1_, TL_1_, and FL_1_. We described two parameters that were calculated for each of three volumes (AL, TL, and FL): lumen volumetric change ratio (LVCR), % (ALVCR, TLVCR, and FLVCR for each corresponding volume); and lumen volumetric expansion rate (LVER), %/year (ALVER, TLVER, and FLVER for each corresponding volume; see Supplementary Equations).

### Fluid dynamics with 4D flow MRI

**Fig. 1:**
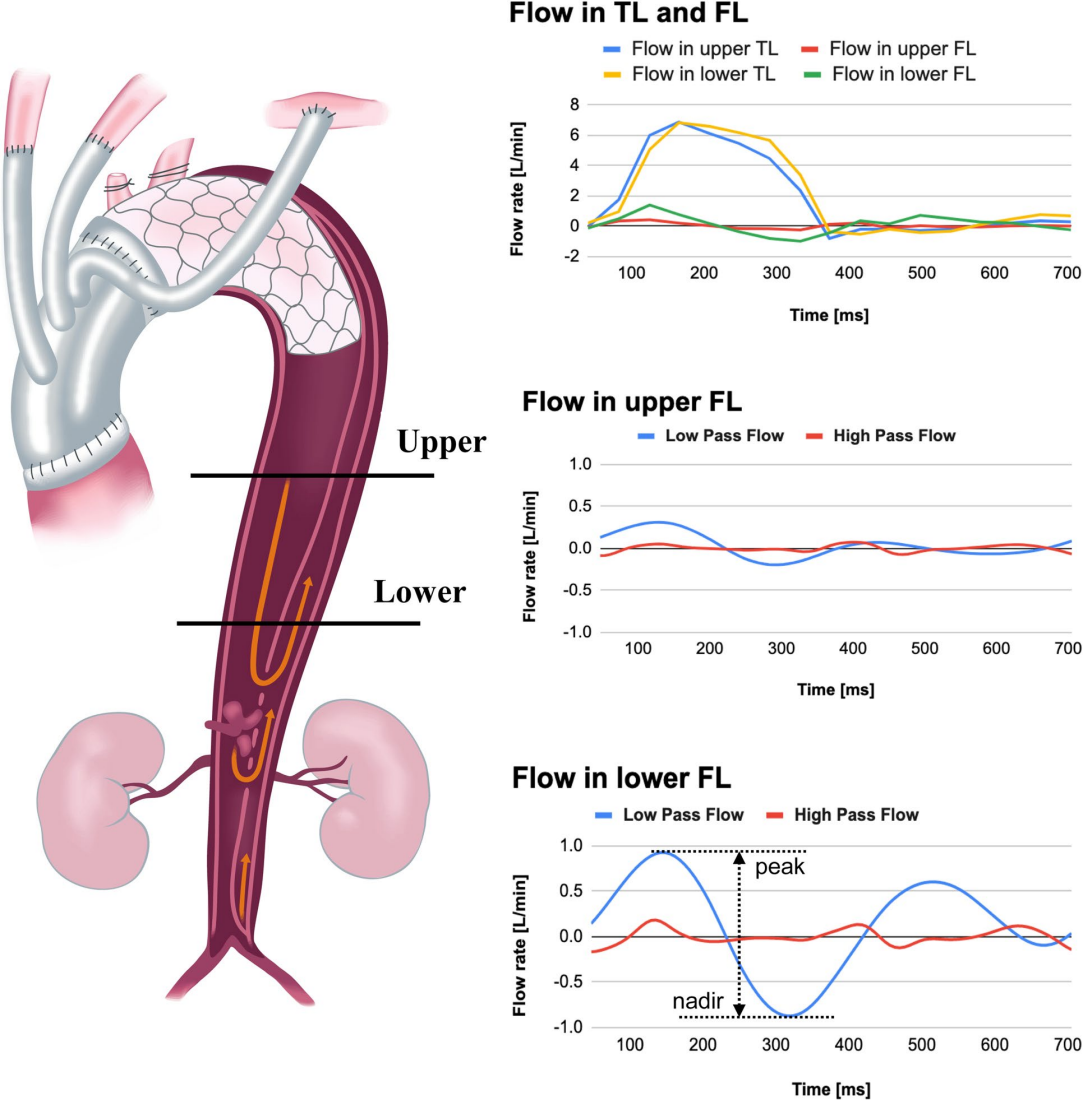
Flow patterns from the pathline in the upper and lower measurement points. *TL* true lumen, *FL* false lumen

Four-dimensional flow MRI was performed using a 3.0-T MRI (Prisma fit 3T, Siemens AG, Munich, Germany) with respiratory motion compensation and electrocardiogram gating with full volumetric thoracic aorta coverage without contrast media. The data were analyzed using specialized software, iTFlow^®^ Version 2.1 [17] (Cardio Flow Design Inc., Tokyo, Japan) by independent investigators. We measured two cross-sectional regions in the target segment: upper and lower regions. The region of interest in the FL had no thrombi or turbulent flow in the upper region. In the lower part, the region of interest in the FL was located above the CA and was not directly affected by re-entry flow. We also measured the flow in the TL of the same region. The flow rate was shown with end-diastole as the origin (0 s). Additionally, blood flow directions were measured with positive numbers showing antegrade flow, and negatives showing retrograde flow. To observe the flow patterns in a cardiac cycle, a low-pass filter was applied to remove high-frequency fluctuating flow. Typical TL and FL flow, as seen in the pathline at the upper and lower measurement points, are shown in Video 1. A flow pattern from the pathline in the upper and lower FL measurement regions is shown in Fig. 1. Typical flow in the lower region of the FL consists of biphasic waves and has a peak and nadir during systole. The flow rate in the upper region is lower than that in the lower region; therefore, these were measured to check for errors. We focused on the flow in the lower region and measured volumetric flow rates (L/min), including net, antegrade, retrograde, peak, and nadir flows.

### Aortic remodeling and negative FL remodeling

Volumetric changes in the segment using LVCR were described in two ways: FL and aortic remodeling. A threshold of 10% was used to define significant changes. Negative FL remodeling was defined as an FLVCR of > 10%. LVCR between − 10% and 10% was stable. According to the method reported by Dohle et al. [16], (1) ALVCR < − 10% (decreased AL) and − 10% ≤ TLVCR (stable TL or increased TL) and (2) − 10% ≤ ALVCR ≤ 10% (stable AL) and TLVCR > 10% (increased TL) were classified as positive aortic remodeling. All other changes were classified as negative aortic remodeling.

### Statistical analysis

Continuous variables are presented as mean (standard deviation [SD]) or median (interquartile range [IQR]), and categorical variables are described as numbers (%). After testing for normality, a comparison of means and medians between the groups was performed using unpaired Student’s *t* test for normally distributed parameters or the Mann–Whitney *U* test for non-normally distributed parameters. Categorical variables were compared using the chi-square or Fisher’s exact tests. Scatter plots and Pearson's correlation analysis were performed to find the 4D flow MRI measures associated with FLVER. All analyses were performed using SPSS version 27 software (IBM Corp., Armonk, NY, USA). A two-tailed p-value of 0.05 was regarded as significant.

## Results

### Patient characteristics

**Fig. 2:**
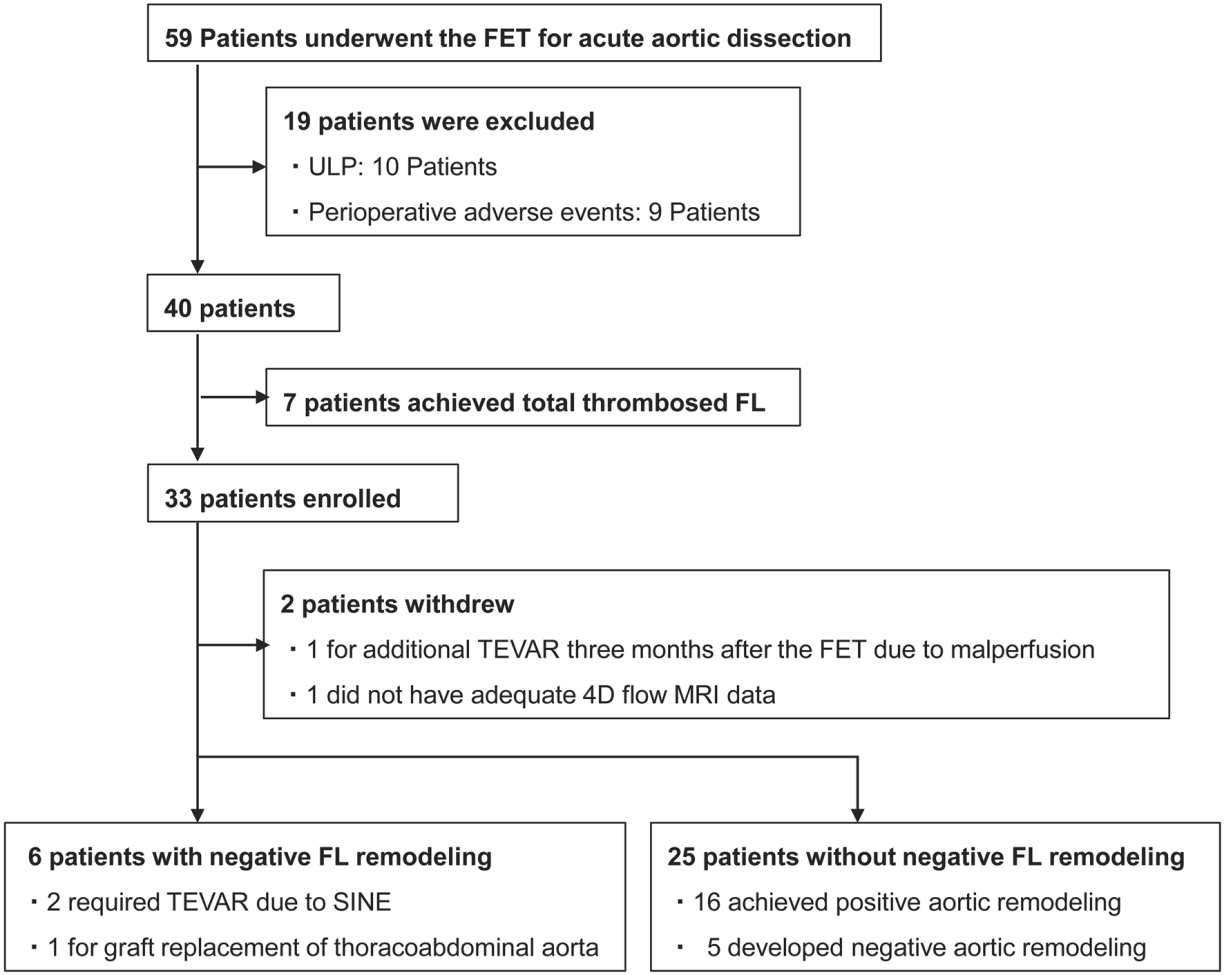
Study profile. *FET* frozen elephant trunk, *ULP* ulcer-like projection, *FL* false lumen, *TEVAR* thoracic endovascular aneurysm repair, *4D flow MRI* four-dimensional flow magnetic resonance imaging, *SINE* stent graft-induced new entry

We performed FET in 59 patients with acute aortic dissection between December 2015 and December 2020. According to our exclusion criteria, 26 patients were excluded, seven of whom had total thrombosis of the FL, achieving aortic remodeling immediately after the FET; finally, 33 patients were included. Two patients withdrew during the early follow-up period. During the observational period, six patients eventually developed negative FL remodeling. Of the 25 patients without negative FL remodeling, 16 achieved positive aortic remodeling, and five exhibited negative aortic remodeling, indicating that only the TL was enlarged, and the aorta was expanded. One patient died of pancreatic cancer, and three required further intervention due to mid-term aortic adverse events. These events included two cases of distal stent graft-induced entry (SINE) with FL enlargement three years after the initial surgery and one case of progressive enlargement of the thoracoabdominal aorta three years after the initial surgery. The former two patients underwent additional thoracic endovascular aneurysm repair (TEVAR), while the latter required graft replacement (Fig. 2). The mean patient age was 56.4 years, 25 (80.6%) patients were male, and 28 (90.3%) underwent FET for Stanford type A aortic dissection. The mean diameter of J Graft FROZENIX^®^ was 27.5 mm, and the median follow-up period was 1.7 years. All patients in the negative FL remodeling group had Stanford type A aortic dissection including one patient with Marfan syndrome. There was no difference in preoperative characteristics between the groups, except for the presence of hypertension. Regarding the number and distribution of re-entries, there were no variations. The re-entries were located in the iliac arteries for most patients (27/31 cases). Eleven (35.5%) patients exhibited negative aortic remodeling, of whom six (19.4%) exhibited negative FL remodeling (Table 1).

**Table 1:** Clinical characteristics and outcomes for clinical characteristics and negative false lumen remodeling

	Overall (*N* = 31)	Negative FL remodeling		
		Yes (*n* = 6)	No (*n* = 25)	*p* value
Demographics				
Age (years)	56.4 [11.0]	48.7 [13.2]	58.2 [9.8]	0.05
Male	25 (80.6)	6 (100.0)	19 (76.0)	0.31
Female	6 (19.4)	0	6 (24.0)	
Stanford type A	28 (90.3)	6 (100.0)	22 (88.0)	1.00
Stanford type B	3 (9.7)	0	3 (12.0)	
Pre-operative comorbidities				
Smoking	7 (22.6)	2 (33.3)	5 (20.0)	0.60
Hypertension	23 (74.2)	2 (33.3)	21 (84.0)	0.03
Diabetes	1 (3.2)	0	1 (4.0)	1.00
CKD	8 (25.8)	1 (16.7)	7 (28.0)	1.00
OCI	0	0	0	
OMI	0	0	0	
Marfan’s syndrome	1 (3.2)	1 (16.7)	0	0.19
J Graft FROZENIX (mm)	27.5 [2.7]	26.7 [2.0]	27.7 [2.8]	0.48
FL_0_ (cm^3^)	90.7 [34.4]	86.0 [31.6]	110.0 [41.4]	0.13
Number of re-entries	3.0 [2.0, 3.5]	3.0 [2.0, 4.5]	3.0[2.0, 3.5]	0.83
Location of the re-entries				
Descending Ao	3 (9.7)	1 (16.7)	2 (8.0)	0.49
CA	10 (32.3)	4 (66.7)	6 (24.0)	0.07
SMA	10 (32.3)	3 (50.0)	7 (28.0)	0.36
RA	16 (51.6)	2 (33.3)	14 (56.0)	0.39
Abdominal Ao	15 (48.4)	2 (33.3)	13 (52.0)	0.65
IA	27 (87.1)	5 (83.3)	22 (88.0)	1.00
Follow-up period (years)	1.7 [1.0, 2.1]	1.9 [1.1, 2.8]	1.7 [1.0, 2.1]	0.79
Negative aortic remodeling	11 (35.5)	6 (100.0)	5 (20.0)	< .01

Data are presented as mean [standard deviation], median [interquartile range], or *n* (%)

*CKD* chronic kidney disease, *OCI* old cerebral infarction, *OMI* old myocardial infarction, *FL*_*0*_ false lumen volume right after the surgery, *CA* celiac artery, *SMA* superior mesenteric artery, *RA* renal artery, *Ao* aorta, *IA* iliac artery

### Volume changes between the two groups

Table 2 presents the volume changes in the target segment. In the negative FL remodeling group, the mean AL volume increased as the TL and FL volumes increased. In contrast, in the group without negative FL remodeling, the average AL volume remained steady with increased TL and decreased FL volumes. Additionally, more than half the patients in the group without negative FL exhibited total thrombosis (Supplementary Table S1).

**Table 2:** Volume change in each lumen over time between the two groups

Volume changes (cm^3^)	Negative FL remodeling			
	Yes (*n* = 6)		No (*n* = 25)	
	Baseline	Latest	Baseline	Latest
AL (AL_0_ → AL_1_)	157.9 [41.7]	216.3 [59.4]	153.6 [40.1]	151.0 [46.9]
TL (TL_0_ → TL_1_)	47.8 [9.8]	68.2 [13.4]	67.6 [21.6]	95.3 [29.9]
FL (FL_0_ → FL_1_)	110.1 [41.4]	148.1 [47.8]	86.0 [31.6]	55.7 [34.3]
Lumen volumetric change ratio (%)				
ALVCR	40.0 [28.3, 48.1]		− 0.2 [− 15.1, 8.1]	
TLVCR	38.2 [31.1, 44.8]		42.2 [24.6, 59.3]	
FLVCR	35.7 [26.3, 45.4]		− 25.8 [− 53.5, − 12.7]	
Lumen volumetric expansion rate (%/year)				
ALVER	15.8 [13.6, 27.2]		− 0.2 [− 7.8, 5.2]	
TLVER	19.8 [7.7, 40.5]		22.6 [10.0, 41.6]	
FLVER	20.1 [13.8, 34.4]		− 17.1 [− 33.4, − 4.8]	

Data are presented as mean [standard deviation], median [interquartile range], or *n* (%)

*FL* false lumen, *AL* aortic lumen, *TL* true lumen, *ALVCR* aortic lumen volumetric change ratio, *TLVCR* true lumen volumetric change ratio, *FLVCR* false lumen volumetric change ratio, *ALVER* aortic lumen volumetric expansion rate, *TLVER* true lumen volumetric expansion rate, *FLVER* false lumen volumetric expansion rate

### Four-dimensional flow MRI analysis for flow volume in the lower waves

**Fig. 3:**
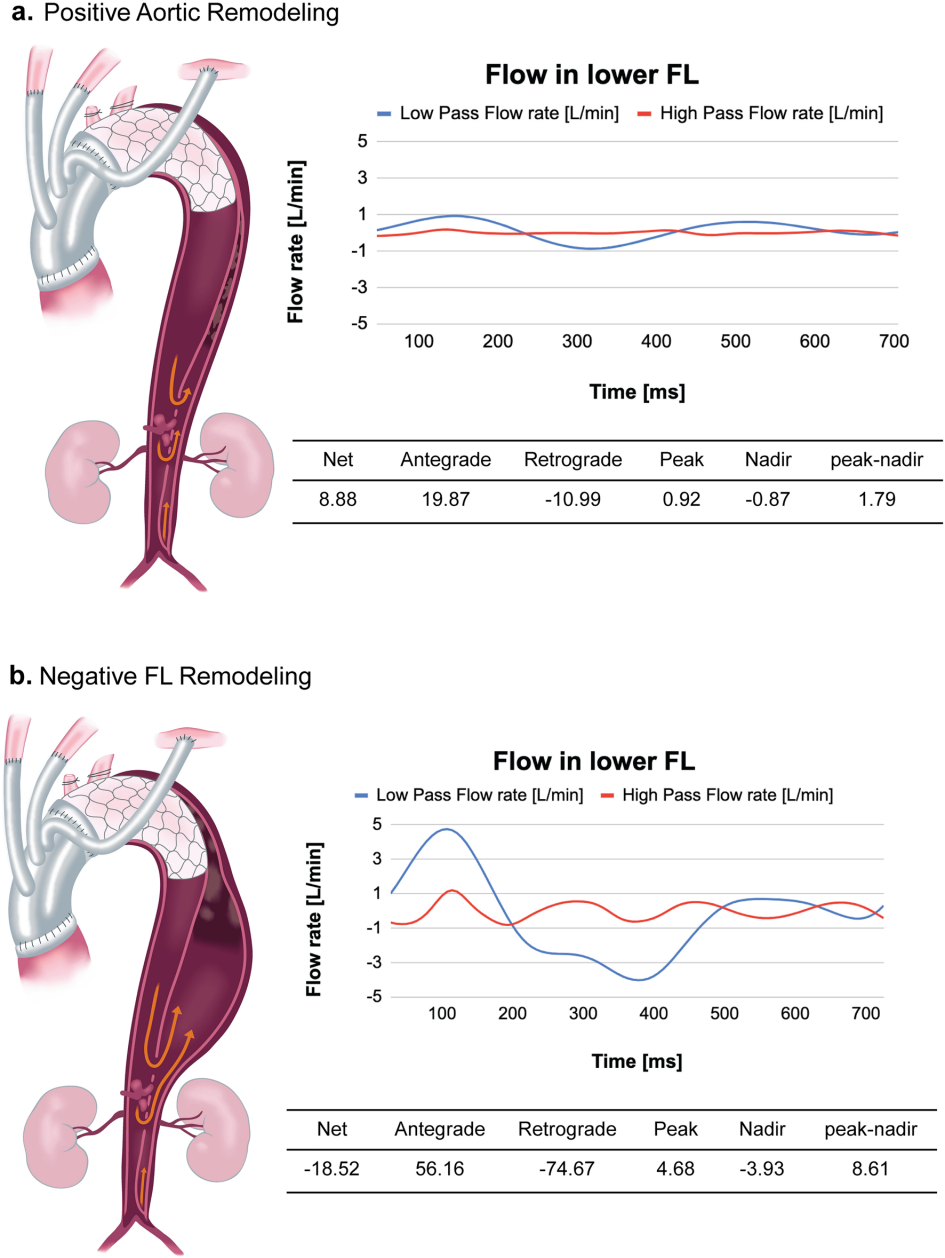
Typical flow pattern and flow rate regarding FL remodeling in the lower FL measurement point. **a** is a positive aortic remodeling, while **b** is a negative FL remodeling. *FL* false lumen

The FL waves at the lower measurement point were higher than those at the upper one, and most FL waves exhibited a biphasic pattern in the systolic phase. Supplementary Figure S1 depicts the flow pattern in all cases and volume change during the observation period. In addition, the FL flow in three patients with re-entry in the descending aorta is shown in Figure S2 and Video 2. Re-entry flow in the descending aorta is considered a part of the early systolic antegrade flow. Figure 3 demonstrates the lower FL waves typical of positive aortic and negative FL remodeling. Table 3 shows that the mean flow rates of net flow and peak flow did not differ between groups, whereas the mean antegrade, retrograde, and nadir flow rates, and the range between the peak and nadir flow were greater in the group with negative FL remodeling.

**Table 3:** Flow volume of the lower wave in the false lumen between two groups

Flow volume (L/min)	Negative FL remodeling		
	Yes (*n* = 6)	No (*n* = 25)	*p* value
Net flow	− 14.49 [14.67]	− 5.94 [12.21]	0.15
Antegrade flow	36.93 [16.34]	17.31 [14.10]	< 0.01
Retrograde flow	− 51.42 [24.36]	− 23.25 [18.32]	0.03
Peak flow	3.01 [1.87]	1.19 [0.87]	0.06
Nadir flow	− 3.52 [1.60]	− 1.57 [1.02]	0.01
Range: Peak–nadir flow	6.53 [3.21]	2.76 [1.70]	0.01

Data are presented as mean [standard deviation]

*FL* false lumen

### Predictor of negative FL remodeling

**Fig. 4:**
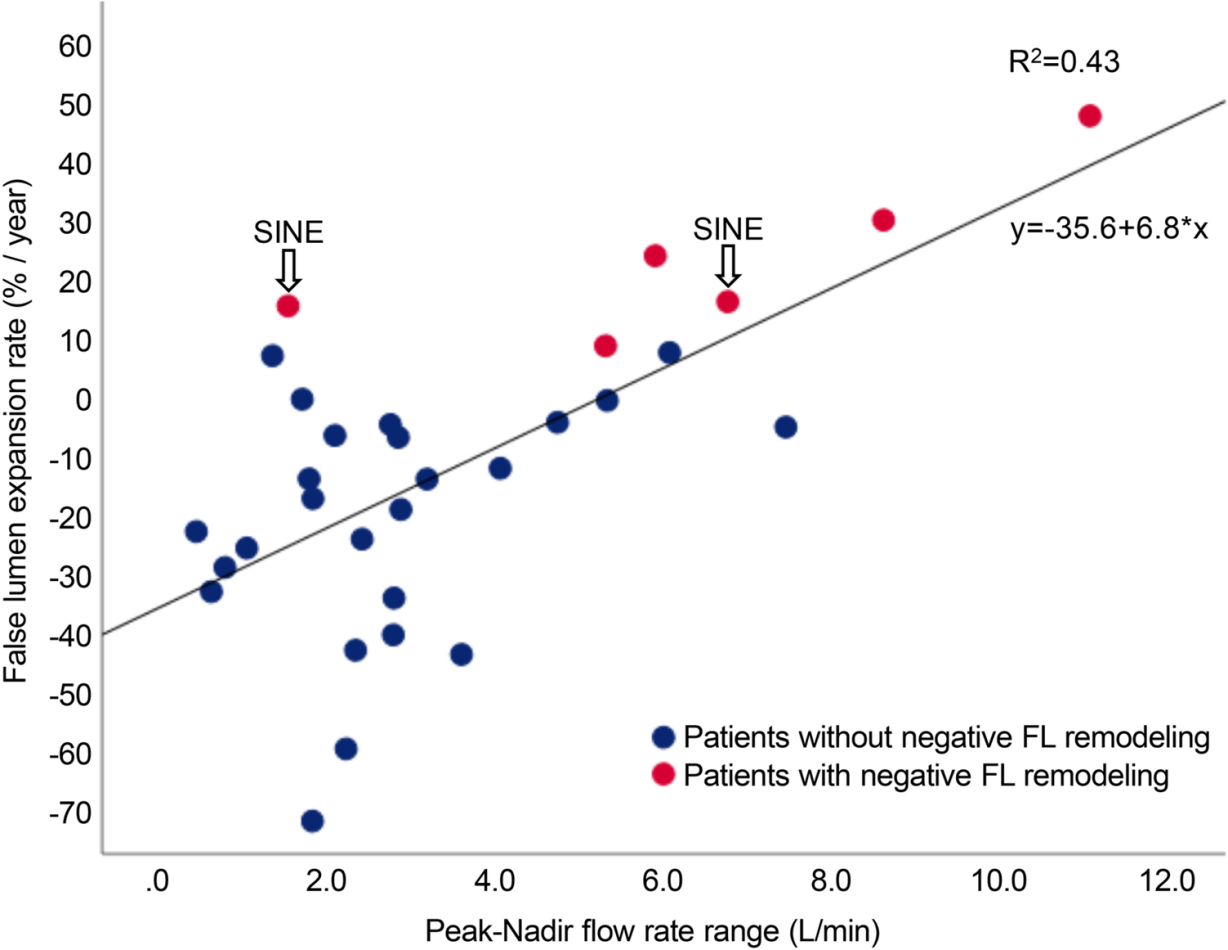
Scatter plot between the peak–nadir flow rate range and false lumen volume expansion rate (FLVER). *FL* false lumen, *SINE* stent graft-induced new entry

Scatter plots and correlation analysis revealed that the flow range between the peak and nadir was associated with FLVER (*R*^2^ = 0.43). Figure 4 shows the linear regression analysis (*β* coefficient = 6.77; *p* < 0.01). Among the patients with negative FL remodeling, those with the lowest flow rate range developed SINE, which rapidly expanded.

## Discussion

This study found that most of the FL flow after FET was biphasic during systole, and the flow range between peak and nadir flow rate of the FL, evaluated from 4D flow MRI analysis, was associated with FLVER in the downstream aorta. Furthermore, flow range can be a predictor of negative FL remodeling.

The FET is associated with excellent early- and mid-term clinical outcomes for acute aortic dissection; this is a result of stent graft implantation with the aim of closing intimal tears in the proximal descending aorta, opening the compressed TL, promoting FL thrombosis, and facilitating positive aortic remodeling [1–3]. Despite the satisfactory success of the treatment up to the proximal descending aorta around the FET with FL thrombosis and positive remodeling, the FL in the downstream aorta remains partially thrombotic or patent, which causes negative aortic or FL remodeling [2, 18, 19]. Previous reports showed a negative aortic remodeling rate of 27% [5] to 35% [8] in the distal thoracoabdominal segments. These patients have a re-intervention risk of 9.6% [4] to 33% [20]. Aortic re-interventions are common mostly after adverse events associated with FET; these events include diameter progression, endoleaks, and distal SINE. In our small series, we had a similar incidence of negative aortic remodeling in the downstream aorta, negative FL remodeling, and re-interventions of 27.5% (11/40), 15.0% (6/40), and 10.0% (4/40), respectively. To avoid re-interventions over time, in some groups, additional stent grafts or bare metal stents were pre-emptively placed after the FET to actively induce FL thrombosis and improve aortic remodeling with a low risk of complications [6, 7, 21]. Placing long-region stent grafts, resulting in occlusion of the thoracic segmental arteries, increases the risk of paraplegia [22]. Therefore, it is crucial to treat only those patients who are likely to develop negative FL remodeling. To date, CT-based predictors of FL enlargement or negative remodeling have been identified as the number of vessels originating from the FL [8] and re-entry locations [9]. However, morphological assessment is limited; this study identified predictive factors by functional evaluation of fluid dynamics using 4D flow MRI. Compared with contrast-enhanced CT, 4D flow MRI does not require radiation or contrast media, making it a non-invasive method. A previous study revealed the feasibility of utilizing flow in TL and FL [23] as predictors of aortic growth rate in patients with uncomplicated type B aortic dissection [24]. In an extensive study of 4D flow MRI for type B dissection, it was reported that the flow pattern and the rate are related to FL enlargement and adverse aortic events [25]. In our study, nearly all patients' FL flow patterns were biphasic during systole (see Supplementary Figure S1). According to our findings, antegrade flow was generated in the TL and FL during early systole, and retrograde flow was generated in the TL and FL during mid-to-late systole. FL flow was considered to be TL flow transmitted through a flap. Some retrograde flow in the FL came from the TL through re-entries. Finally, the reflected antegrade flow was observed in the FL during diastole (Video 1). It is thought that FL enlargement occurs when there is high retrograde flow, including re-entry flows; however, in this study, both the retrograde and antegrade flow rates were significantly higher in the group with negative FL remodeling compared to the other group. We speculate that the large wavelength of biphasic flow inhibits the generation of blood clots in the FL. The dynamic changes of flow, coupled with directional shifts, generate shear stress in the FL wall, thereby interfering with FL remodeling over time. These findings suggest that managing the flow generated by flap movement and re-entry flow can prevent FL enlargement. We explain why we focused on a specific parameter, the flow range between peak and nadir flow rates. One might argue that utilizing measured flow rates, such as net, antegrade, and retrograde flow rates, would offer a more suitable approach than relying on the calculated flow range between peak and nadir flow rates to assess the relationship with FLVER. However, the measured flow rates were notably low, potentially leading to velocity errors at certain measurement points owing to the background phase errors inherent in 4D flow MRI [26]. This flow range is, therefore, the most accurate representation of the widths of antegrade and retrograde flows. The components of the flow range include changes in the FL flow rate due to flap vibration and re-entry flow rate. The biphasic flow may be predominantly due to flap elasticity, potentially influenced by the TL flow rate and blood pressure. The retrograde component of the biphasic flow is expected to be more pronounced with higher re-entry numbers and larger re-entry sizes. Using existing modalities, flap oscillation width is measured with four-dimensional CT, and the number and size of re-entries are measured through multiplanar reconstruction of CT data, which often leads to inter-rater errors. Therefore, it would be clinically valuable to use 4D flow MRI to focus only on FL flow. In addition, correlating the number and size of re-entries and flow information is challenging. Future research should explore the correlation between these parameters and the range of flow rates. The flow pattern and flow range results from this study can provide a rationale for additional TEVAR. If a stent graft is implanted in an area with re-entries, the FL will thrombose, whereas even when an additional TEVAR is performed in an area without re-entries, the FL is frequently thrombosed. As demonstrated in this study, placing a stent graft in the TL presumably prevents biphasic flow from propagating into the FL through the flap. Taken together, additional TEVAR should be performed only in cases where this flow range is large. Finally, it is necessary to mention that two cases of SINE were reported during follow-up among the patients that developed negative FL remodeling. FET size selection was determined through measurements; thus, stent graft oversizing cannot be the cause of SINE. In the first case, a small flow range was observed, and the FL gradually shrank until the onset of SINE. Subsequently, the FL underwent rapid expansion after the onset of SINE. The underlying reason for the SINE was believed to be the spring-back force, an occurrence independent of the flow range. In the second case, the flow range was relatively large, and the FL expanded until SINE onset. As previously reported [27], the flap was moved upward as a result of FL expansion, which may have caused SINE due to mechanical stress on the distal end of the FET.

## Limitations

First, a multivariate analysis could not be performed because of the small number of patients. Confounding factors were considered well in advance as far as possible. Second, the artifacts associated with 4D flow MRI and figuring out the timing to perform the imaging are also limitations. The most significant artifact of 4D flow MRI is motion artifact due to patient movement. Since 4D flow MRI takes much longer (30–40 min) than a routine MRI, some patients move during prolonged imaging, leading to missing diastolic data. Studies aimed at shortening the examination time can help make 4D flow MRI more common. Most 4D flow MRIs were performed within two weeks to one month after surgery, and before discharge from the hospital. This period is the “subacute phase” of the chronicity of dissection. However, there were a few cases where imaging was performed beyond one month after surgery due to time-consuming rehabilitation, including transfers or adjusting the examination date. Although the lack of uniformity in the timing of imaging may be problematic from a research perspective, it still falls under the same “subacute phase” if it is within three months after surgery. The condition of the flap may change over time, which may affect the flow pattern of the FL. It would be advisable to perform a 4D flow MRI within one month after surgery to ensure the reproducibility of this study. Third, all CT and 4D flow MRI analyses were performed manually. To avoid measurement bias, CT and MRI analyses were performed by separate teams, and the results were withheld until the end of the analysis. Finally, TL flow rate evaluation was not included in our study methodology. The non-negative FL remodeling group included five patients with negative aortic remodeling, indicating that the TL expanded more than the FL and the aortic volume was increased by 10%. The median peak-to-nadir flow range in those patients was low (2.1 L/min). While the analysis of TL flow might be essential to comprehend this phenomenon, our study focused exclusively on FL flow analysis because the predominant literature focuses on aortic negative remodeling attributed to FL enlargement.

## Conclusion

We found that FL flow after the FET procedure is biphasic during systole, and the range between peak and nadir flow rates may play an important role as a hemodynamic predictor of FL expansion rate and negative FL remodeling. Further research is required to validate these findings, but they are expected to be applicable clinically along with other morphologic predictors for FET patients who may require additional procedures.

### Supplementary Information

Below is the link to the electronic supplementary material.

Supplementary file1 Video 1: Typical true and false lumen flow as seen in the pathline at the upper and lower measurement points. Blue represents true lumen flow, while red represents false lumen flow. (MPG 5616 KB)

Supplementary file2 Video 2: Pathlines of the cases with re-entry in the descending aorta. **A** has a large re-entry and **B** and **C** have smaller ones. Blue represents true lumen flow, while red represents false lumen flow. (MPG 17470 KB)

Supplementary file3 (DOCX 3621 KB)

## Data Availability

The data that support the findings of this study are available from the corresponding author, Y. T., upon reasonable request.
