# Supplementary material for: Hemodynamic predictors of negative false lumen remodeling after frozen elephant trunk for acute aortic dissection
Source: Gen Thorac Cardiovasc Surg. 2023 Nov 10;72(6):376–86. doi: 10.1007/s11748-023-01984-x (PMC11127806; doi:10.1007/s11748-023-01984-x)
Supplement: Supplementary file 3 — Supplementary file3 (DOCX 3621 KB) [file 11748_2023_1984_MOESM3_ESM.docx]

**Supplementary Equations**

1. Lumen volumetric change ratio (LVCR) = (LV_1_ -LV_0_)/ LV_0_.

(ⅰ) Aortic lumen volumetric change ratio (ALVCR) = (AL_1_ -AL_0_)/ AL_0,_

(ii) True lumen volumetric change ratio (TLVCR) = (TL_1_-TL_0_)/ TL_0,_

(iii) False lumen volumetric change ratio (FLVCR) = (FL_1_ -FL_0_)/ FL_0._

2. Lumen volumetric expansion rate (LVER) = LVCR / follow-up years x 100

(ⅰ) Aortic lumen volumetric expansion rate (ALVER) = ALVCR / follow-up years x 100

(ii) True lumen volumetric expansion rate (TLVER) = TLVCR / follow-up years x 100

(iii) False lumen volumetric expansion rate (FLVER) = FLVCR / follow-up years x 100

**Supplementary Table S1.** Degree of thrombosis in the FL between the two groups

|  | Negative FL Remodeling | |
| --- | --- | --- |
| Degree of thrombosis in the FL | Yes (n = 6) | No (n = 25) |
| Patent | 1 (16.7) | 2 (8.0) |
| Partial thrombosis | 5 (83.3) | 7 (28.0) |
| Total thrombosis | 0 | 7 (28.0) |
| Obliterated | 0 | 9 (36.0) |

FL, false lumen

**
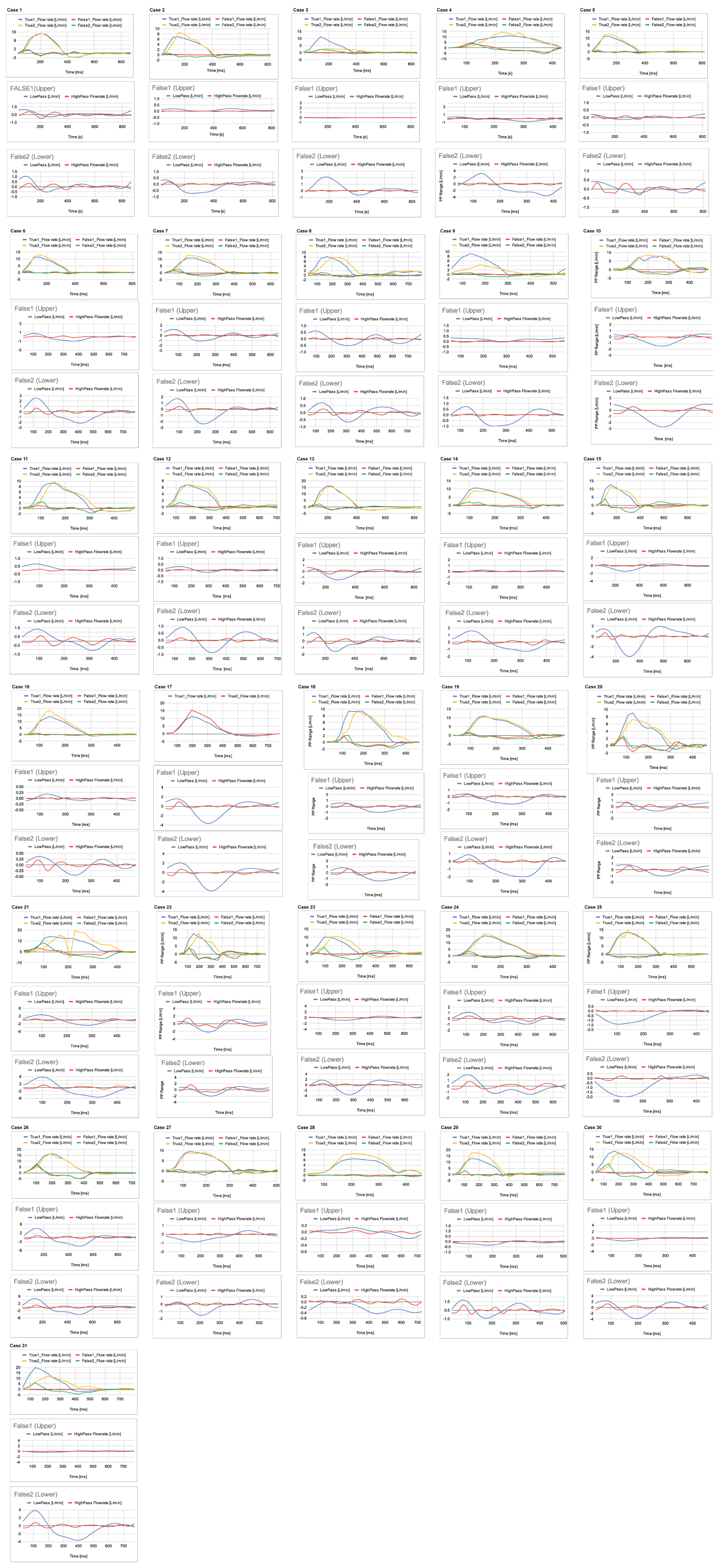
**

**Supplementary Fig. S1**: Flow patterns of all the patients


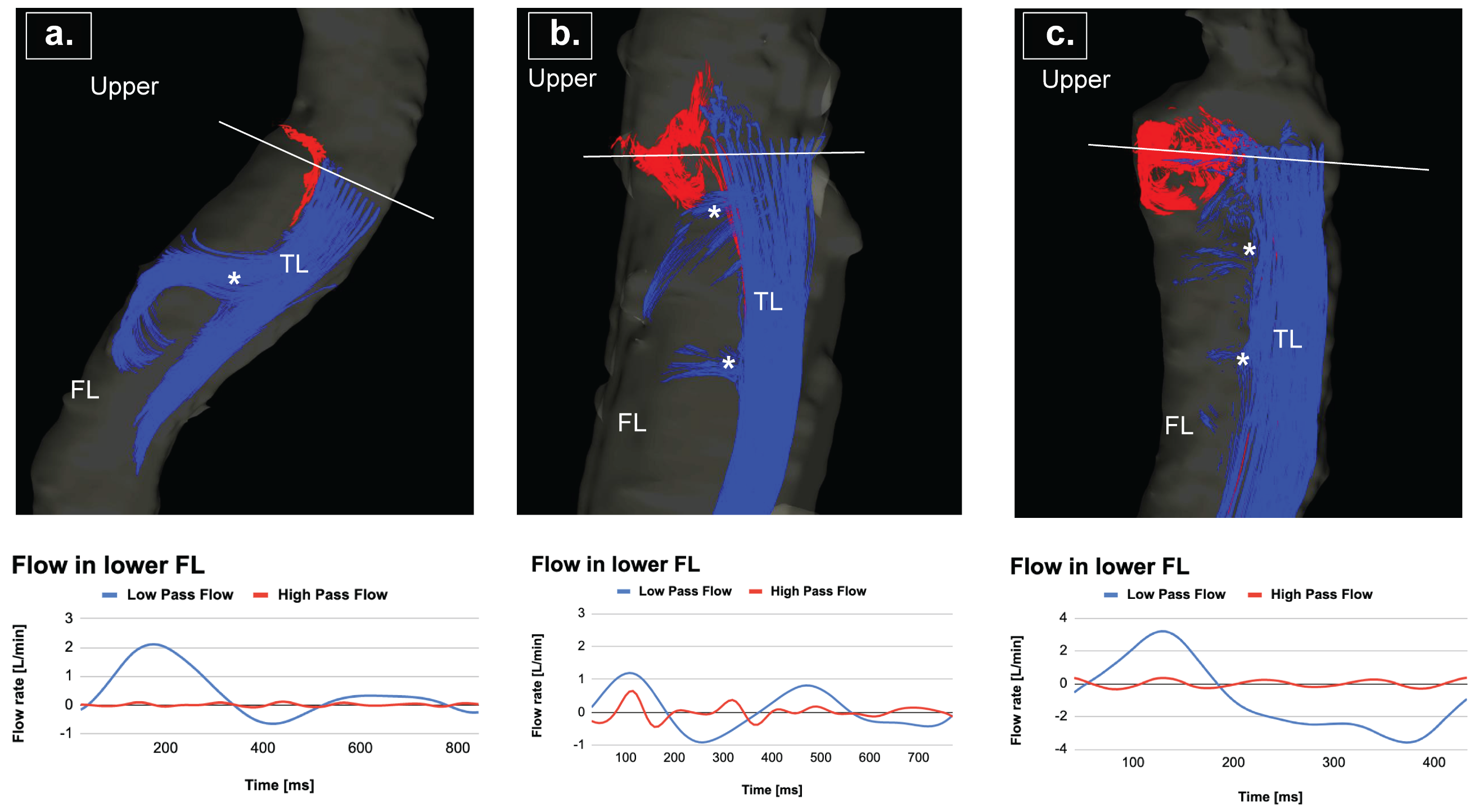


**Supplementary Fig. S2**: Path lines and flow patterns in the lower measurement point with re-entry into the descending aorta. (a) shows a case with large re-entry. (b) and (c) show small re-entries in the descending aorta. Asterisks indicate re-entries.
